# Brazilian Butt Lift Safety and Florida Legislature: What You Should Know, How You Can Help

**DOI:** 10.1093/asjof/ojad041

**Published:** 2023-05-06

**Authors:** Jeffrey M Kenkel, Max Polo, Pat Pazmiño, Onelio Garcia

The authors are pleased to present an expert video roundtable discussion on gluteal augmentation with fat, known widely as the Brazilian Butt Lift, with a focus on the patient safety bills currently being voted on by the Florida legislature (Video). The discussants include a panel of leading experts in aesthetic surgery, and the video roundtable was recorded live at The Aesthetic Meeting 2023 in Miami, FL, USA. The panelists provide an overview of the current challenges, areas of research, and education being undertaken to improve patient safety in gluteal augmentation. As the recording took place, Florida legislators were preparing to vote on bills that would regulate how gluteal fat grafting should be performed and whether plastic surgeons, nurse, or physician's assistants should perform this procedure. The message of these panelists is singular and clear: only board-certified plastic surgeons should perform gluteal augmentation with fat in its entirety. The discussants believe that what happens in Florida represents both an opportunity and a framework for how the rest of the United States will respond to the questions posed about gluteal augmentation.

The overarching goal of this video roundtable is to protect patients and to place their safety at the forefront of the discussion. They review the significance of using ultrasound during gluteal augmentation with fat to allow for clear visual confirmation that the cannula is not in the muscle during fat graft injection. This has been shown and published in the *Aesthetic Surgery Journal*^1–3^ and *ASJ Open Forum*^4^ and is directly related to patient outcomes and mortality rates. This research has helped save lives. It is imperative that government regulators stand with the board-certified plastic surgeons to improve safety measures that will lead to better patient outcomes and a continued decrease in mortality rates not only in Florida, but worldwide.

The panelists issue a call to action to all viewers. Visit the following link to let Florida legislators know that you stand with patient safety and that only surgeons should do surgery: mia.one/house.

## Supplementary Material

ojad041_Supplementary_Data
